# *Carum carvi* and *Ormenis multicaulis* Extracts: Chemical Analysis and In Vivo Experiments to Evaluate the Synergistic Analgesic and Anti‐Inflammatory Effects

**DOI:** 10.1002/cbdv.202501286

**Published:** 2025-09-11

**Authors:** El Mahdi Wakrim, Karima Raoui, Hamid Kabdy, Jawad Laadraoui, Mehdi Ait Laaradia, Chafik Terrafe, Sami Mnif, Brahim Bouizgarne, Stefania Garzoli, Abderrahman Chait

**Affiliations:** ^1^ Laboratory of Plant Biotechnology “Biotechnologies Végétales”, Faculty of Sciences University Ibn Zohr Agadir Morocco; ^2^ Laboratory of Pharmacology, Neurobiology, Anthropology and Environment, Department of Biology, Faculty of Sciences, Semlalia University Cadi Ayyad Marrakesh Morocco; ^3^ Laboratory of Physiopathology, Genetic Molecular and Biotechnology, Faculty of Sciences, Aïn Chock Hassan II University Casablanca Morocco; ^4^ Higher Institute of Nursing Professions and Health Techniques Ministry of Health and Social Protection Beni Mellal Morocco; ^5^ Laboratory of Molecular and Cellular Screening Processes Centre of Biotechnology of Sfax University of Sfax Sfax Tunisia; ^6^ Department of Chemistry and Technologies of Drug Sapienza University Rome Italy

**Keywords:** analgesic activity, anti‐inflammatory activity, antioxidant power, chemical composition, methanolic extracts, phenolics, synergy

## Abstract

The purpose of the present investigation was to determine the effects of combination of *Ormenis multicaulis* dry flower methanolic extracts and *Carum carvi* seed methanolic extracts on pain and inflammation, compared to their use separately. High‐performance liquid chromatography (HPLC) analysis of methanolic extracts showed the presence of phenolic compounds. Extracts of the two plant samples showed antioxidant properties demonstrated by 2,2‐diphenyl‐1‐picrylhydrazyl (DPPH) activity scavenging and ferric reducing antioxidant abilities, significantly higher than controls (quercetin and butylated hydroxyanisole [BHT]). During the pharmacological study, the extracts from a mixture of 50% of each plant sample were orally administered to mice animals and analgesic and anti‐inflammatory effects were registered. The evaluation of the analgesic activity showed a synergistic effect of the mixture from the two plant extracts, compared to each plant extract applied alone or to the control. Peripheral analgesic effect was demonstrated, and dual (central and peripheral) analgesic effect was confirmed by inhibition of pain in the last phase of the formalin test. Moreover, methanolic extracts of *O. multicaulis* and *C. carvi*, when applied either alone or in combination, showed significant anti‐inflammatory. Synergistic effects of the two methanolic extracts could represent a promising potential alternative medicine for the treatment of inflammation and pain.

## Graphical abstract

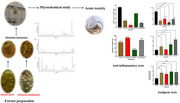

## 1 Introduction

Several diseases are accompanied by sensations of unpleasant or harmful inflammation and pain. Because of their negative effects, the prescription of common anti‐inflammatory and analgesic drugs may be restricted. Inflammation is considered a response that causes heat, redness, edema, and discomfort in response to infectious, chemical, and physical agents such as radiation, poisons, bacteria, and caustic chemicals. On the other hand, the definition of pain is a sensation ranging from minor discomfort to excruciating suffering. Pain can be localized, such as in an injury, or it can be more widespread [1]. Traditional and complementary health care can be a valuable resource for identifying and generating plant‐based medicines with lesser drawbacks. Natural compounds derived from plants or microbia possess biological activities, including anti‐inflammatory, analgesic, and antibacterial properties [2, 3]. Due to the relevance of herbal medicines to human health, new plant‐derived pharmaceuticals being used as alternative or complementary treatments for pain and inflammation management are needed [3, 4]. Among medicinal herbs, caraway (*Carum carvi*) and chamomile (*Ormenis multicaulis*) are of the most traditionally used in Morocco. *C. carvi*, belonging to the Umbelliferae family, known as Persian cumin or caraway, was used as a spice and a medicinal herb since antiquity. Fruits of caraway are well‐known as a stimulant of milk production in lactating mothers, to stimulate menstruation, and as diuretic [5, 6, 7]. It is also used for healing hyperglycemia, hypertension, and heart and renal diseases [8]. *C. carvi* seeds were used in folk medicine as breath re‐fresher and expectorant for helping to improve appetite and digestion, especially in children. Seeds possess antispasmodic, carminative, antiflatulence, astringent and are used as laxatives in treatment of gastrointestinal disorders such as diarrhea, dyspepsia, hysteria, flatulent indigestion, colic, and dyspeptic symptoms [9, 10]. *C. carvi* also possesses lactiferous and diuretic properties, antioxidant and anticancer properties [5, 11, 12]. It also possesses antibacterial, antifungal, and anti‐aflatoxinogenic effects [13, 14, 15]. Furthermore, molecules from seeds, such as essential oil, fatty oil, oleoresins, and carvone, are widely used in the food and medicinal industries [9]. In addition, *C. carvi* extracts were reported to possess analgesic activity [10]. *O. multicaulis* originates from East Asia and Europe, being widely used as herbal medicine since antiquity. It possesses a broad spectrum of biological properties, including anti‐allergic, antimicrobial, and analgesic. Moroccan chamomile, also known as simple‐leaved chamomile, is a tall spontaneous annual plant with fragrant white–yellow flowers that grows in sandy soils near the Atlantic coast of Morocco. Moroccan chamomile is prevalent in the Gharb area and is mostly utilized for the extraction of essential oil [16]. Morocco is the primary provider of this species’ essential oil to the worldwide market, and the oil is utilized in the perfume and medicinal industries [16]. It was reported that varieties of chamomile possess antimicrobial, anti‐mold, antiviral, anti‐amoebic, antispasmodic, anti‐inflammatory, antioxidant, anti‐allergic, analgesic, anti‐hypertensive, and anticancer activities in addition to its beneficial effects as hepatoprotective and nephroprotective. Chamomile has also beneficial effects on central‐nervous‐system‐related disorders, such as sleep disorders, epilepsy, and Alzheimer [17, 18]. Medicinal properties that various herbs possess are well‐documented; the synergy of their combinations is also well‐documented [19]. It was reported that combinations have the advantage over individual plant‐based as they could result in potential synergistic interactions between herbs, which can enhance beneficial therapeutic effects or mitigate potential adverse effects of each individual herb [19, 20]. Although beneficial effects of *C. carvi* and *O. multicaulis* were reported [5, 6, 7, 8, 17, 18], there are no available reports on synergistic effects of *C. carvi* and *O. multicaulis* combinations as antioxidant and as efficient in reducing pain (analgesic effects) and inflammation (anti‐inflammatory effects). Thus, the aim of this investigation was to determine how methanolic extracts of *C. carvi* and *O. multicaulis* as well as their combination, attenuate pain and inflammation in different animal models.

## 2 Results and Discussion

### 2.1 Total Phenolic Compounds, Flavonoids, and Tannins of *C. carvi* and *O. multicaulis*

Total phenolic compounds content (TPC), total flavonoids content (TFC), and total condensed tannins content (TTC) of *O. multicaulis* and *C. carvi* extract are summarized in Table 1. Results of measurements in *O. multicaulis* dry flowers methanolic extracts showed 10.96, 7.07, and 2.57 mg/100 g DM, respectively, for TPC, TFC, and TTC. Results also showed that in *C. carvi* seed methanolic extracts, all those compounds are present with higher concentrations compared to those of *O. multicaulis*. Indeed, *C. carvi* methanolic extracts showed 50.18, 24.50, and 8.41 mg/100 g DM, respectively, for TPC, TFC, and TTC.

**TABLE 1:** Total phenolics (TPC), total flavonoids (TFC), and total tannins content (TTC) of *Carum carvi* and *Ormenis multicaulis* methanolic extracts mean ± SEM.

Methanolic extract	Total polyphenols (mg GAE/100 g DM)	Total flavonoid (mg CE/100 g DM)	Total tannins (mg CE/100 g DM)
*Carum carvi*	50.18 ± 1.02	24.50 ± 0.35	8.41 ± 0.22
*Ormenis multicaulis*	10.96 ± 1.05	7.07 ± 0.56	2.57 ± 1.66

### 2.2 High‐Performance Liquid Chromatography (HPLC) Analysis of Phenolic Compounds

**FIGURE 1:**
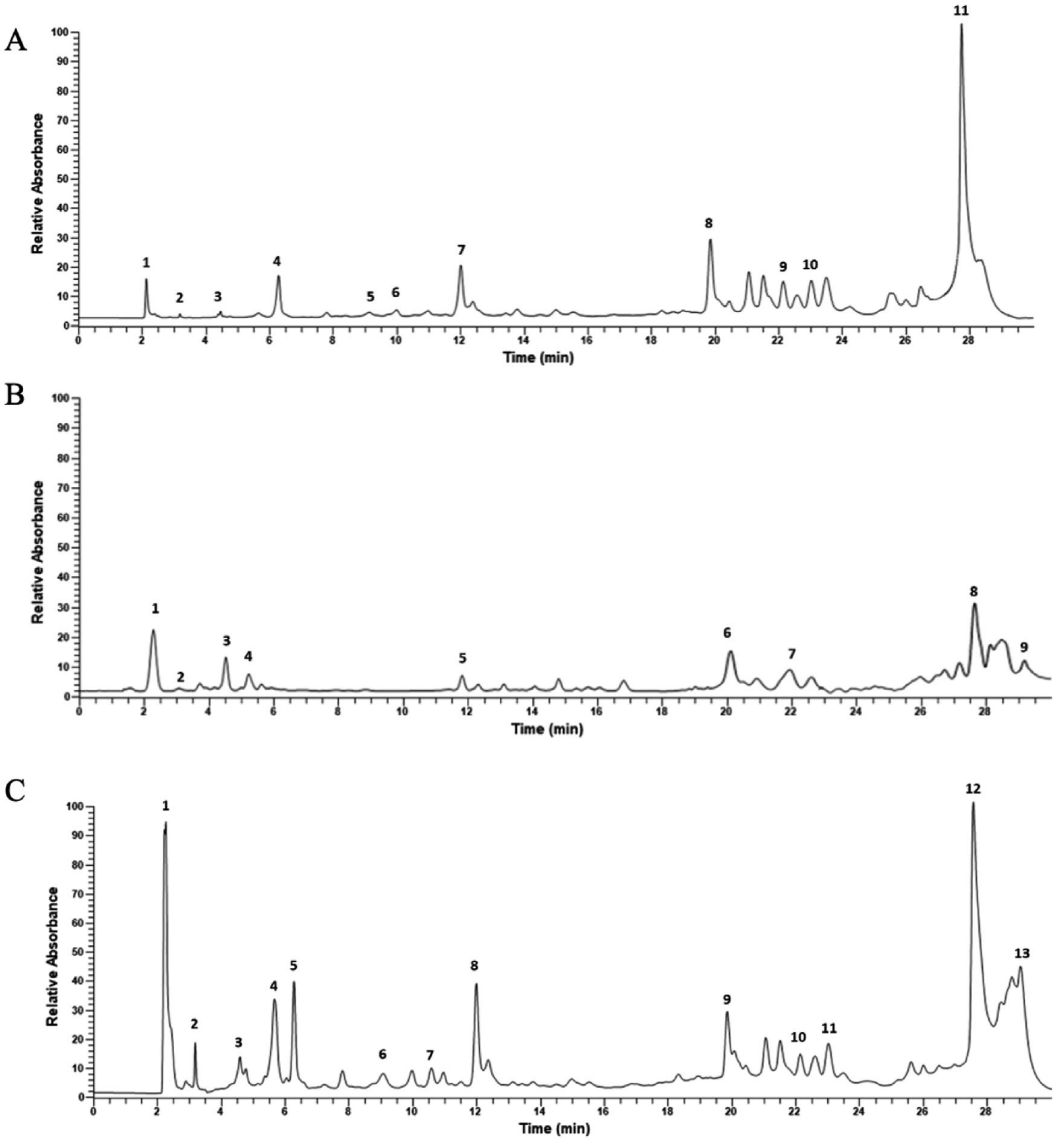
HPLC chromatogram for the main phenolic compounds identified in the methanolic extract of (A) *Carum carvi*. 1: gallic acid; 2: catechic acid; 3: ferulic acid; 4: caffeic acid; 5: cinnamic acid; 6: vanillic acid; 7: quinic acid; 8: syringic acid; 9: luteolin; 10: kaempferol; 11: quercetin. (B) *Ormenis multicaulis*. 1: gallic acid; 2: catechic acid; 3: ferulic acid; 4: *p*‐hydroxybenzoic; 5: quinic acid; 6: syringic acid; 7: luteolin; 8: quercetin; 9: apigenin. (C) Mixture of *C. carvi* and *O. multicaulis*. 1: gallic acid; 2: catechic acid; 3: ferulic acid; 4: *p*‐hydroxybenzoic; 5: caffeic acid; 6: cinnamic acid; 7: vanillic acid; 8: quinic acid; 9: syringic acid; 10: luteolin; 11: kaempferol; 12: quercetin; 13: apigenin.

HPLC analysis of methanolic extracts of *O. multicaulis, C. carvi*, and of their mixture shows the presence of gallic acid (GA), catechin acid, ferulic acid, quinic acid, syringic acid, luteolin, quercetin in the two extracts (Figure 1A,B, and Table 2). Results also showed a slight increase in the concentrations of other compounds in *O. multicaulis* dry flowers methanolic extract, particularly for *p*‐hydroxybenzoic acid and apigenin (respectively, 5.15 and 5.61 µg mL^−1^). In addition, *C. carvi* seed methanolic extracts showed the presence of other compounds, such as caffeic acid at a concentration of 24.43 µg mL^−1^, followed by kaempferol at a concentration of 11.01 µg mL^−1^ DM, with the presence of cinnamic acid and vanillic acid at concentrations of 3.30 and 3.51 µg mL^−1^, respectively (Table 2). Of 13 phenolic compounds, apigenin and *p*‐hydroxybenzoic acid were not detected in *C. carvi* seed methanolic extracts, whereas caffeic, cinnamic, and vanillic acids and kaempferol were not detected in *O. multicaulis* dry flower methanolic extract. The mixture has the advantage of showing the presence of all the phenolic compounds present in each of the plant samples.

**TABLE 2:** Concentrations of the main phenolic compounds identified in the *Carum carvi*, *Ormenis multicaulis*, and their mixture methanolic extracts.

Phenolic compounds	Concentrations of extract (µg mL^−1^)		
	*C. carvi*	*O. multicaulis*	Mixture
Apigenin	0.00	5.61	2.17
Caffeic acid	24.34	0.00	12.87
Catechin acid	2.03	1.70	1.24
Cinnamic acid	3.30	0.00	1.24
Ferulic acid	2.61	3.79	2.79
Gallic acid	19.30	22.68	21.69
*p*‐Hydroxybenzoic acid	0.00	5.15	3.57
Kaempferol	11.01	0.00	5.09
Luteolin	9.51	5.50	8.20
Quercetin	65.77	32.87	50.02
Quinic acid	26.95	8.40	18.38
Syringic acid	22.66	12.18	18.12
Vanillic acid	3.51	0.00	1.13

### 2.3 Acute Toxicity of *C. carvi* and *O. multicaulis* Extracts

The acute toxicity test in mice did not record any behavioral changes in mice at investigated doses (0.5–5 g kg^−1^ body wt.). Furthermore, *C. carvi* seed and *O. multicaulis* dry flower methanolic extracts did not show any sign or symptom of toxicity during the observation period of 24 h after the administration of the extracts. No significant modification of organ weight or body weight. Administered doses did not lead to any death 14 days following the administration of the extracts. As a result, LC_50_ was higher than 5 g kg^−1^ body wt.

### 2.4 Antioxidant Capacity of *C. carvi* and *O. multicaulis* Extracts

**FIGURE 2:**
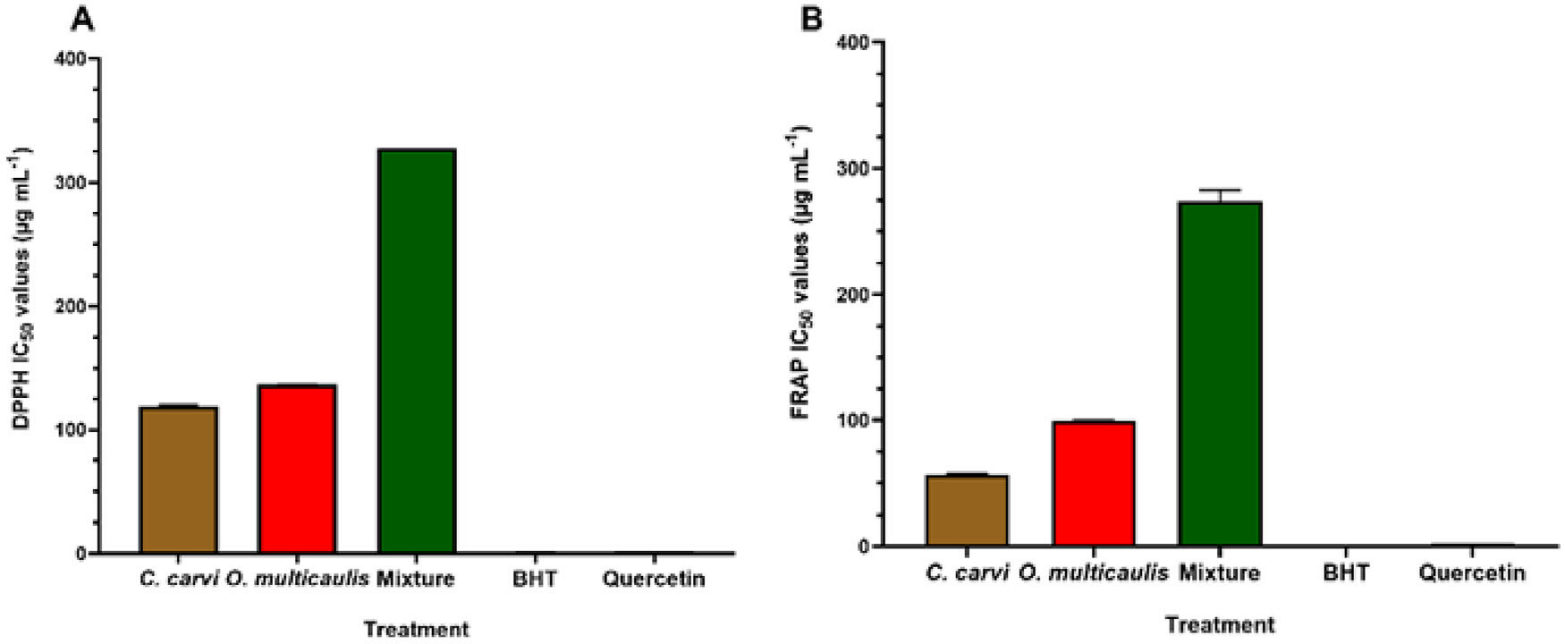
Antioxidant effects of methanolic extract of *Carum carvi*, *Ormenis multicaulis*, and their mixture methanolic extracts, measured as DPPH‐IC_50_ (µg mL^−1^) (A) and FRAP‐IC_50_ (µg mL^−1^) (B), compared to the reference antioxidants quercetin and butylated hydroxyanisole (BHT).

The antioxidant activity of *C. carvi* and *O. multicaulis* extracts was evaluated in vitro using two complementary assays: 2,2‐diphenyl‐1‐picrylhydrazyl (DPPH) and ferric reducing antioxidant power test (FRAP test) assays. The concentrations that resulted in 50% inhibition (IC_50_) are shown in Figure 2. The antioxidant activities were compared with that of quercetin and butylated hydroxyanisole (BHT). DPPH‐IC_50_ values were 119.1 and 136.44 µg mL^−1^, whereas FRAP‐IC_50_ values were 56.69 and 99.33 µg mL^−1^, respectively, for *C. carvi* and *O. multicaulis* methanolic extracts. For the two antioxidant assays, DPPH‐ and FRAP‐IC_50_ values of *O. multicaulis* were higher than those of *C. carvi*. Interestingly, methanolic extracts of the mixture showed highest DPPH‐ and FRAP‐IC_50_, 327.98 and 274 µg mL^−1^, respectively, representing 2.4 and 2.75 times higher values than those of *O. multicaulis* used alone.

### 2.5 Analgesic Properties of *C. carvi* and *O. multicaulis* Extracts

#### 2.5.1 Writhing Test

**FIGURE 3:**
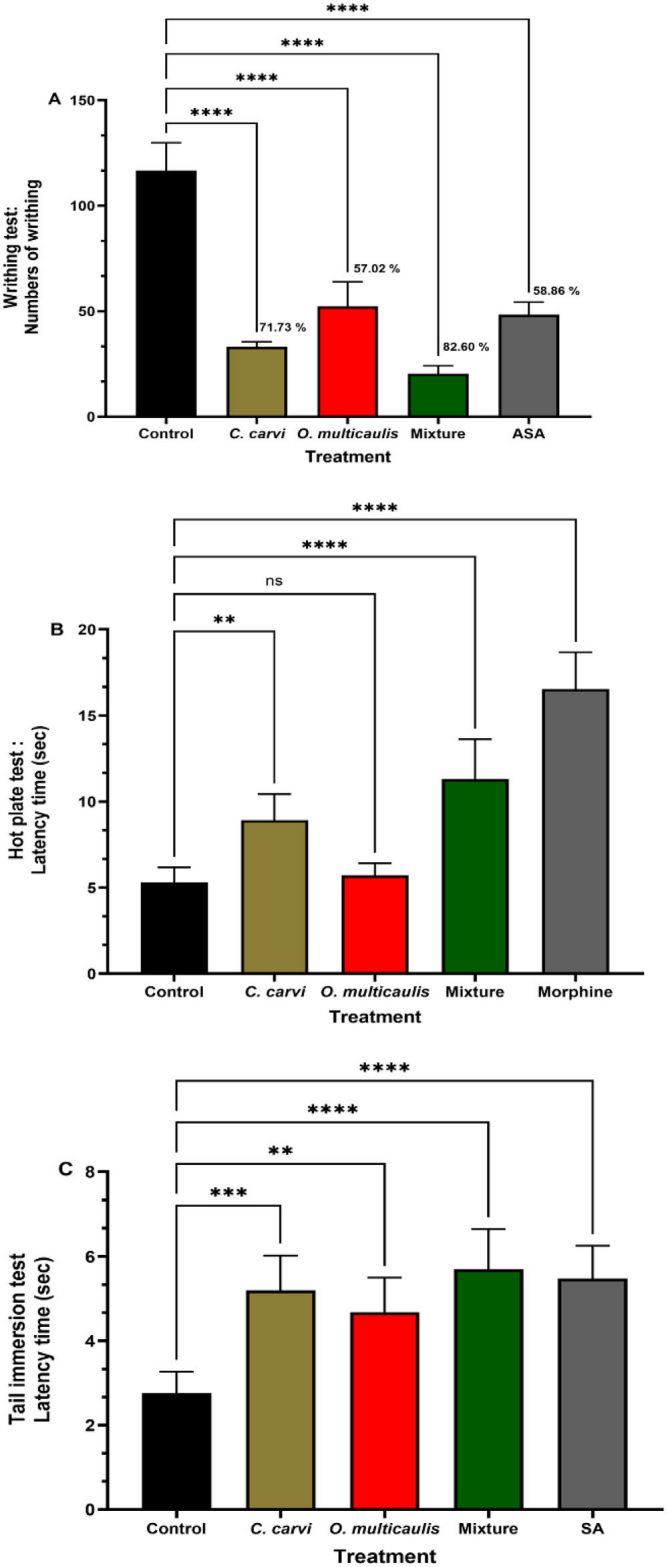
Antalgic effect of the methanolic extract of *Carum carvi, Ormenis multicaulis*, and their methanolic mixture extracts during (A) number of contortions during the writhing test (acetic acid), (B) latency time in hot plate test, and (C) latency time in tail immersion test. Data are presented as mean ± SEM. Values above graphs are percentage of inhibition (%). Asterisks represent significant differences at *p < 0.05; **p < 0.01; ***p < 0.001 compared to control group; +*p* < 0.05; +++*p* < 0.001 compared to mixture group.

Assay aiming to evaluate the analgesic properties of *O. multicaulis*, *C. carvi* extracts, and extracts from their mixture on pain was performed by using intraperitoneal injection of a 0.6% acetic acid‐induced contortion test in mouse model. Number of abdominal writhes was about 120 in mice treated with acetic acid. This number was only 49 when mice were treated with acetylsalicylic acid (ASA) 30 min before acetic acid injection, leading to a decrease in writhes number and an antinociceptive activity of 58.86%. The number of abdominal contortions was around 34 and 51, respectively, in *C. carvi* and *O. multicaulis* treated groups subsequently injected by acetic acid. It significantly decreased, respectively, by 71.73% and 57.02% compared to the control group. In group administered with the extracts from the mixture of *O. multicaulis* and *C. carvi* and subsequently injected by acetic acid, the number of writhes did not exceed 21, showing a stronger response than each of the plants *O. multicaulis* and *C. carvi* used separately and even greater than the reference molecule ASA. The mixture showed a reduced number of writhes by 82.6% (Figure 3A).

#### 2.5.2 Hot Plate Test

In this test, the methanolic extracts of *O. multicaulis* and *C. carvi* and extracts from their mixture time of latency in mice during the hot plate assay were compared with the control and a reference drug, morphine. Morphine presented high analgesic activity (Figure 3B). In control group, time of latency was only 5.30 s, whereas morphine administration resulted in a time of latency of 11.31 s. Compared to control, *O. multicaulis* response latency was higher (5.71 s), whereas *C. carvi* methanolic extracts resulted in a higher response latency time (8.91 s), representing an increase of 1.08 and 1.68 fold, respectively. In comparison to the effects of each extract administered alone, the mixture of *C. carvi* and *O. multicaulis* resulted in the highest response latency (16.53 s), which corresponds to a 3.12 fold increase compared to control, even higher than morphine (11.31 s, 2.13 fold increase).

#### 2.5.3 Tail Immersion Test

Tail immersion test was performed in order to test the analgesic activity of the extracts from and from their mixture. Results showed that in comparison to control, all treatments resulted in a significant increase in the latency time. Control latency time was 2.76 s, whereas *C. carvi* and *O. multicaulis* showed values of 5.20 and 4.68 s, respectively, significantly different from the control. Mixture treatment resulted in higher latency value (5.70 s), significantly different from the control and higher than latency time obtained with each plant extract taken separately, whereas the positive treatment (morphine) led to similar latency time (5.48 s), not significantly different from the mixture (Figure 3C).

#### 2.5.4 Formalin Test+

In both phases of the formalin‐induced nociception model, extracts of *O. multicaulis*, *C. carvi*, and their combination significantly reduced paw licking time in treated mice compared to the control group. During the neurogenic phase, the analgesic responses were 38%, 50%, and 64% for *C. carvi*, *O. multicaulis*, and their mixture, respectively. In the inflammatory phase, *O. multicaulis*, *C. carvi*, and the combined extracts decreased licking time by 43%, 30%, and 47%, respectively. Regarding the reference drugs, ASA exhibited a strong antinociceptive effect only in the second phase, whereas morphine showed significant effects in both phases (Table 3).

**TABLE 3:** Effect of *Carum carvi*, *Ormenis multicaulis*, and their mixture methanolic extracts in the formalin‐induced nociception, compared to acetylsalicylic acid and morphine.

	Neurogenic phase		Inflammation phase	
Groups/Treatment	Licking time (s)	Inhibition %	Licking time (s)	Inhibition %
**Control (NaCl 9 ‰)**	122.93 ± 2.45	—	70.87 ± 4.81	—
***Carum carvi***	75.40 ± 1.13	38.66^*^	46.74 ± 3.23	30.04^**^
***Ormenis multicaulis***	60.50 ± 3.16	50.78^*^	39.84 ± 2.46	43.78^**^
**Mixture**	43.40 ± 2.76	64.66^**^	37.15 ± 5.20	47.58^**^
**Acetylsalicylic acid**	67.61 ± 1.04	45.01^**^	23.81 ± 2.35	66.40^***^
**Morphine**	25.59 ± 1.10	79.18^***^	36.61 ± 1.12	48.34^**^

^*^ *p* < 0.05, ^**^ *p* < 0.01, and ^***^ *p* < 0.001 compared to control group.

### 2.6 Anti‐Inflammatory Properties of *C. carvi* and *O. multicaulis* Extracts (Xylene Test)

**FIGURE 4:**
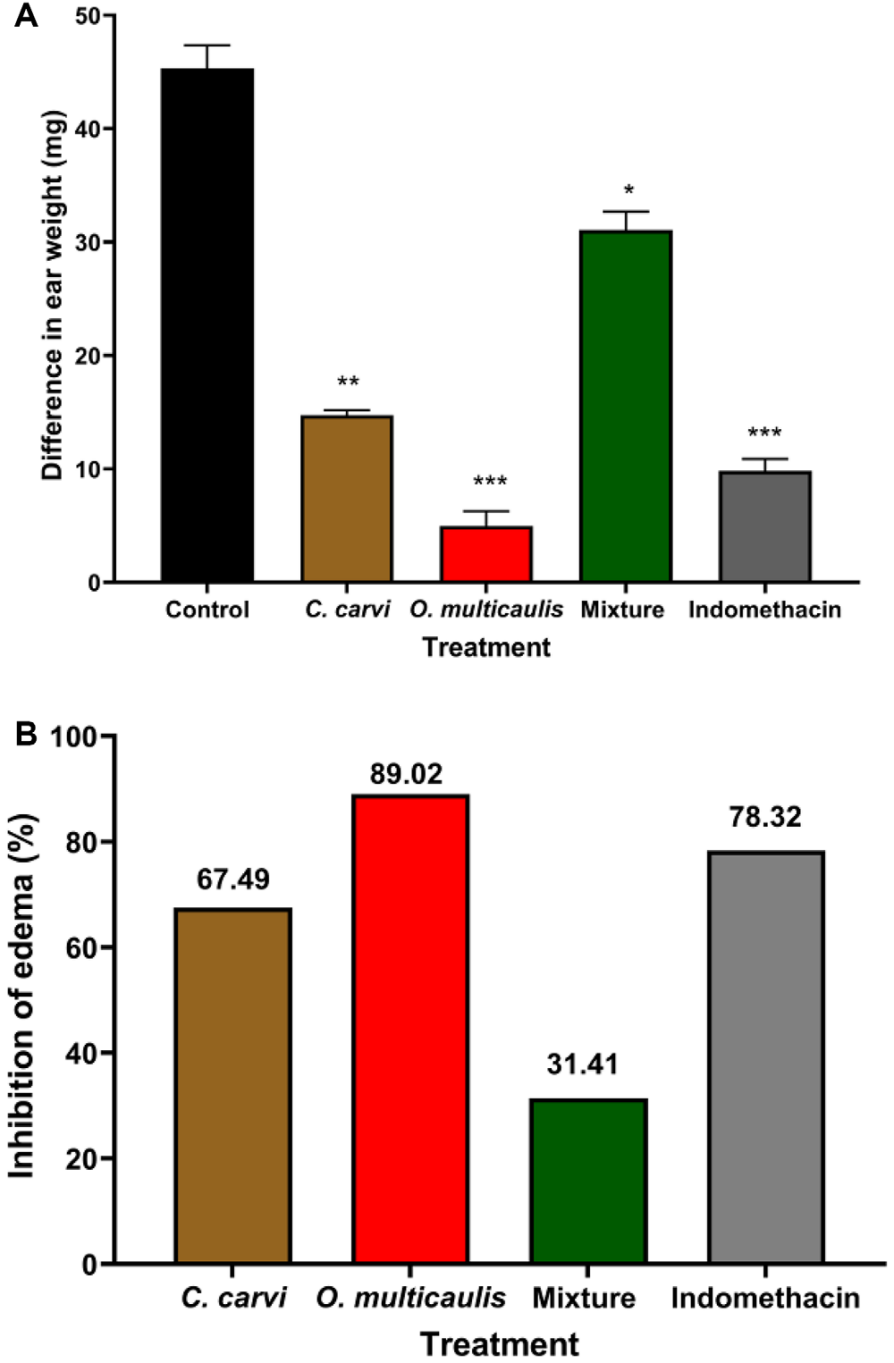
Anti‐inflammatory activity of *Carum carvi*, *Ormenis multicaulis*, and their mixture methanolic extracts during xylene‐induced ear edema test. (A) Mean difference in ear weight (mg), (B) registered inhibition of edema (%). Values represent mean difference in ear weight (mg). Asterisks represent significant differences at *p < 0.05, **p < 0.01, and ***p < 0.001 as compared to negative control group.

In the xylene‐induced ear edema test, the negative control group (xylene) exhibited increased ear edema, whereas the positive control (indomethacin) and *O. multicaulis* methanolic extract significantly reduced edema by 78.32% and 89.02%, respectively, 15 min after injection (*p* < 0.001). Methanolic extracts of *C. carvi* and the mixture containing *C. carvi* also demonstrated notable edema reduction potential, with decreases of 67.49% (*p* < 0.01) and 31.41% (*p* < 0.05), respectively, compared to the control group (Figure 4). However, the mixture did not result in greater inhibition, achieving only 31.41% reduction.

According to Mosby's Medical Dictionary [21], pharmacological synergy refers to the joint action of two or more molecules or drugs in such a way that one enhances the effect of the other, resulting in a combined effect greater than that achieved by either molecule alone at equivalent doses, or producing effects unattainable by any safe dose of either molecule individually, or both. Nowadays, the synergistic effects of herbal combinations are gaining increasing attention from researchers. Several combinations have been reported as successful in therapy [20, 22, 23], whereas others are controverted, weakly supported, or still to be proved [24, 25]. This is mainly due to the presence of multiple components in herbs, causing interactions that are often unpredictable and complicated [24]. Plants were used for pain and inflammation therapy. Assayed plants include chamomile [18, 26] and caraway [9, 10, 11]. However, combinations of *O. multicaulis* and *C. carvi* are not yet investigated. Thus, the purpose of this study was to compare the effects of *O. multicaulis* and *C. carvi* methanolic extracts, either alone or in a mixture, on some pharmacological effects, especially pain and inflammation, using the mouse animal model. Phytochemical results showed that extracts revealed a higher content of phenolic and flavonoid compounds, such as GA, catechin acid, ferulic acid, quinic acid, syringic acid, luteolin, and quercetin. In our investigation, the acute toxicity test results, performed on groups of six Swiss mice, using orally administered methanolic extracts of *C. carvi* and *O. multicaulis* at 0.5–5 g kg^−1^ body wt. p.o. [27, 28], showed no behavioral changes and no symptoms of toxicity during 24 h. In addition, no changes in organ or body weights were recorded in the treated group 14 days following the administration of the extracts. LC_50_ was demonstrated to be higher than 5 g kg^−1^ body wt., suggesting non‐toxic effects at this dose. Similar results were found for saffron, orally administered to Swiss albino mice [29]. Our results suggest antinociceptive effects of the two extracts. Moreover, we demonstrated synergistic effects when combination of methanolic extracts of *C. carvi* and *O. multicaulis* was administered. Antinociceptive effects were demonstrated in various plants [1, 10, 26, 30, 29, 31, 32, 33, 34, 35, 36, 37, 38]. The effect of methanolic extracts of *C. carvi*, *O. multicaulis*, and their combination was tested on mice model in the writhing test. Results showed that the abdominal writhes were strongly inhibited by each of the extracts applied alone. Chaves et al. [26] showed a reduced nociception induced when using a purified extract from chamomile tea. Similar responses were shown when saffron (*Crocus sativus*) stigma hydro‐ethanolic extract was orally administered to Swiss albino mice model [29] and in a more recent work by Araújo et al. [32], where it was demonstrated that aqueous extract of *Cannabis sativa* roots possesses in vivo effect of reducing oxytocin‐induced abdominal contortions. Interestingly, *C. carvi* and *O. multicaulis* mixture extracts resulted in a higher writhing reduction in mice. It could be concluded that the mixture possesses a synergistic antalgic effect at peripheral level. The mechanism of pain occurring after intraperitoneal injection of acetic acid was explained by Hunskaar and Hole [30]. In addition, acetic acid induces an increase in peritoneal fluid by activating vascular permeability following the secretion of prostaglandins, histamine, and serotonin [36]. It has also been described that the response of abdominal constriction is induced by the activation of peritoneal receptors and the prostanoid mediators involved [39]. In fact, the increased levels of PGE2 and PGF2 in the peritoneal fluid and the production of lipoxygenase inhibitors were reported in some previous studies [37]. However, they would not act on the opioid system in the modulation of pain. The findings of the current investigation showed that ASA causes significant inhibition of acetic acid‐induced pain, which is consistent with earlier results indicating that the assay is responsive to non‐steroidal anti‐inflammatory drugs (NSAIDs) [40]. Thus, the pain‐relieving and anti‐inflammatory action of the mixture of *O. multicaulis* and *C. carvi* extracts seems to be mediated by the inhibition of lipoxygenase and cyclooxygenase activity, either by the liberation of cytokines like TNF‐a, interleukin‐8, interleukin‐1, or by residual peritoneal macrophages and mast cells [41]. The hot plate test is used to study the involvement of vanilloid receptors in the analgesic effects of opioids. Vanilloid receptors, in particular the potential transient vanilloid receptor 1 (TRPV1) receptor, play a role in the perception and modulation of pain. Opioids can interact with these receptors to produce analgesic effects. By using specific agonists or antagonists of TRPV1 receptors, researchers can study the contribution of these receptors to opioid‐induced analgesia in the hot plate test. In our investigation, extracts of *C. carvi* and *O. multicaulis* and extracts from the mixture increased latency of the mice on the hot plate compared to the control. Compared to control, in response to *O. multicaulis* extract administration, latency was slightly higher than control. However, *C. carvi* methanolic extracts resulted in a higher response, whereas the mixture resulted in the highest response. The mixture led to a latency higher than morphine. Those effects indicate the possible implication in central analgesic effect. Similar central, antinociceptive effect in response to thermal stimuli was showed when saffron (*C. sativus*) stigma hydro‐ethanolic extract was orally administered to Swiss albino mice model [29]. The results obtained showed that extracts from *C. carvi* have higher analgesic effects than *O. multicaulis* extracts. Interestingly, extracts from the mixture of *O. multicaulis* and *C. carvi* provoked a significant higher analgesic effect on the central pain compared to each plant extract administered alone. This effect was even higher compared to the positive control morphine. This result suggests a high potential of the mixture as mitigator at the central pain level, causing increased latency of the response to the thermal stimulus.

According to tail immersion experiments, the two extracts of *C. carvi* and *O. multicaulis* resulted in a prolonged latency time compared to control. Significant differences were found between control and the extracts from the two plants. In addition, the mixture resulted in a synergistic effect, similar to salicylic acid (SA) effect; indeed, no significant difference was found between the mixture SA. The induced effects leading to higher latency time resulted in a relative heat pain alleviating effect. This could be due to action on opioid receptors involved in the central mechanism of analgesic activity, and this mechanism is known to elevate the pain threshold toward heat [42]. Chamomile was reported to possess nociceptive beneficial effects. Using formalin, test and Swiss mice, a highly substituted 4‐*O*‐methyl‐glucuronoxylan, derived from chamomile tea, intraperitoneally injected at 30 mg kg^−1^ body wt. [26], showed a 96% reduced nociception compared to the control.

In order to confirm analgesic effect mentioned in previous model tests, formalin test was used to examine possible dual central and/or peripheral mechanisms [29]. This test induces paw licking following an injection of formalin solution, which leads to a biphasic response. Indeed, the first phase that occurs directly after the injection of formaldehyde stimulates the C fibers and releases the P substances and the bradykinin. The second phase causes the inflammatory pain caused by the release of mediators such as histamine, serotonin, prostaglandins, and bradykinins that stimulate the nociceptors [43]. In our investigation, *C. carvi* and *O. multicaulis* extracts and extracts from their mixture significantly reduced paw licking time in treated mice groups. In both neurogenic and inflammatory phases, these effects were higher than reference drugs ASA and morphine during the neurogenic phase. The results of this test allowed us to assume that the synergy of the mixture of *C. carvi* and *O. multicaulis* would act on the two phases by reducing the cumulative time of licking. The mixture possesses an analgesic effect on the central pain by acting mainly on the early phase. Similar dual (central and peripheral), antinociceptive effect in response to formalin was showed when saffron (*C. sativus*) stigma hydro‐ethanolic extract was orally administered to Swiss albino mice model [29]. These results are consistent with those reported by Seddighfar et al. [10] in a work using formalin test with a mixture of *Malva sylvestris*, *Medicago sativa*, and *C. carvi* hydroalcoholic extracts, administered to male Wistar rats.

Our results also showed antioxidant effects of methanolic extract of *C. carvi, O. multicaulis*, and their mixture methanolic extracts IC_50_ compared to the reference antioxidants quercetin and BHT. *C. carvi* showed a DPPH‐IC_50_ of 136.44 µg mL^−1^, higher than *O. multicaulis* (119.10 µg mL^−1^). Similarly, *C. carvi* showed higher FRAP IC_50_ of 99.33 µg mL^−1^ compared to *O. multicaulis* (56.69 µg mL^−1^). The mixture possesses the highest DPPH‐IC_50_ of 1295.33 µg mL^−1^ and a ferric reducing power FRAP‐IC_50_ of 274 µg mL^−1^, significantly higher than values of each plant extract alone. This led us to conclude that mixtures do not present any synergy for higher radical scavenging properties. Ghannay et al. [44] studied antioxidant properties of *C. carvi* L. essential oil and found that it exhibited antioxidant activity with DPPH value of 15 mg mL^−1^, higher than the values of DPPH‐IC_50_ (136.44 µg mL^−1^) found in this investigation. Phenolic extract from *C. carvi* flowers revealed a higher DPPH‐IC_50_ of 2.7 µg mL^−1^ [45]. Polar extracts from German chamomile (*Matricaria recutita* L.) root and flowers revealed DPPH‐IC_50_ values ranging from 13 to 57 µg mL^−1^ [46], lower than those found in our investigation for *O. multicaulis* (119.10 µg mL^−1^). Synergistic antioxidant activities were reported by works by Hajlaoui et al. [47], where *Coriandrum sativum* essential oils showed less antioxidant activity (DPPH‐IC_50_ 38.83 mg mL^−1^) than essential oils from the seeds of *C. carvi* (DPPH‐IC_50_ 34.00 mg mL^−1^. However, mixture showed higher antioxidant activity (DPPH‐IC_50_ of 19.00 mg mL^−1^). All those results suggest the importance of the plants origin, the species used, plant parts used for extraction (flowers, tepals, seeds, roots), the nature of samples used in a mixture, plant sample size, the nature of compounds used as antioxidants (bulk or purified extracts), and their concentrations and extraction procedure (essential oils, hydraulic, or alcoholic extracts) for radical scavenging assay. Caraway ethanolic extract was able to prevent the cancer progression by modulating the antioxidant system [48]. These antioxidative effects of *C. carvi* were attributed to its principal compound, carvone, a terpene found in seed essential oils [47, 49]. Regarding the anti‐inflammatory tests, the xylene test induced inflammation in the ear with the appearance of four major clinical signs, including redness, heat, pain, and edema. Following application of xylene to the ear, statistically significant weight increases were found as a result of the acute inflammatory response. These increases in ear weight have been used as precious indicators of anti‐inflammatory effects [50].

In this study, the weight of the right ear decreased for all the groups of animals, either those treated with the mixture or the extracts alone, the result of which is statistically significant. The mixture did not show synergy. This inhibition could, however, be considered evidence that the mixture of the both extracts used in this research still holds a level of beneficial effect on the acute inflammatory response, though not as higher than effect of each plant extract used separately. Plant extracts proved their effectiveness in alleviating xylene‐induced ear inflammation. The role of plant phenolic compounds in the anti‐inflammatory effects was reported by many researchers. It was demonstrated that flavonoids from leaves of *Juniperus sabina* significantly inhibited xylene‐induced ear edema in mice in a dose‐dependent manner [51]. It was also demonstrated that alcoholic extracts from *Pituranthos scoparius*, propolis, *Dianthi herba*, and *Pogostemon cablin* alleviated inflammatory effects in mice following xylene treatment [33, 52, 53, 54].

Phytochemical analysis of methanolic extracts from both plants revealed the presence of phenolic compounds, flavonoids, saponins, sterols, terpens, alcaloides, and tanins (only total phenolics, flavonoids, and tannins data are shown). Similar results were found by Chauhan et al. [55] in caraway seeds and by Chauhan and Aishwarya [56] in German chamomile (*M. recutita*) flower. Phenolic and flavonoid compounds are well known as anti‐inflammatory and analgesic active compounds [38, 51, 57]. Antioxidative and anti‐inflammatory effects of chamomile were suggested to be due to phenolic compounds such as apigenin and its glycosides and flavonoids, particularly luteolin [58, 59, 60, 61, 62]. Best antioxidative results were obtained when methanol was used to extract phenolic compounds from caraway seeds [63].

Our results showed that quercetin and quinic acid were major phenolic compounds in methanolic extracts of caraway, whereas GA and quercetin were major phenolic compounds in methanolic extracts of chamomile. In the mixture, GA and quercetin were also major compounds. Sotiropoulou et al. [64] found that the major phenolics of German chamomile (*Matricaria chamomilla*) aqueous extracts were rutin, ferulic acid, chlorogenic acid, and apigenin‐7‐*O*‐glucoside. Caraway seed methanolic extract has been reported to show the presence of caffeic acids, quercetin, and kaempferol [65]. It was reported that German chamomile (*M. chamomilla* L.) and Roman chamomile (*Chamaemelum nobile* L.) contain various components, namely, flavonoids, terpenoids, and coumarins, which are responsible for their medicinal properties as an anti‐inflammatory, antioxidant, and analgesic herbs. Its antioxidative and anti‐inflammatory effects were linked to a reduced reactive oxygen species (ROS) level, particularly H_2_O_2_, and decreasing prostaglandin and cyclooxygenase activity [18]. It was also reported that chamomile beneficial effects on central‐nervous‐system‐related disorders such as sleep disorders, epilepsy, and Alzheimer [18] were probably due to the flavonoid and apigenin [18, 60]. Our results suggested that the changes in the content of total phenolic and flavonoid might contributed greatly to the analgesic, anti‐inflammatory, and antioxidant synergism in the extract obtained from mixture of *O. multicaulis* and *C. carvi*. Therefore, the effect of extracts on acute inflammation is probably due to inhibition of prostaglandin production or their bio‐transport [34, 35]. Synergistic effects in the mixture could be due to their higher total phenolic compound concentration compared to each plant extract used alone.

Previous works have shown that synergy could result from a complex interaction between single ingredients with different pharmacological functions, such that one ingredient enhances the therapeutic effect of another active ingredient [66, 67] or via coalistic combinations, so all the ingredients involved are inactive individually but become active in combinations [68]. It appears that similar interactions between the ingredients are produced in the combination tested in this study, and therefore this combination exhibited synergy in the activities measured. The mixture of the plant extracts tested in low doses in this work can have remarkable anti‐inflammatory and analgesic effects. The combination of these two extracts can be a rational substitute for conventional therapies that cause adverse effects in patients.

## 3 Conclusions

Methanolic extracts of *O. multicaulis* and *C. carvi* showed a group of biochemical compounds and a diversity of phenolic compound molecules. The two species showed synergistic analgesic and anti‐inflammatory properties of extracts from their mixture. The extract from their combination may act through the central inhibitory pathways, probably due to a reduction of prostaglandin production. These extracts have the potential to be utilized as medications to treat pain and inflammation.

## 4 Experimental Section

### 4.1 Material and Plant Material

Chamomila (*O. multicaulis*) was collected in February 2020 in Ouezzane region (34°48′ N, 5°35′ W, Morocco), and caraway (*C. carvi*) was collected from the province of Ourika, Sti Fadma village (30°43′48″N, 6°33′0″W, Morocco). The botanical identity of the plants has been confirmed by Prof. Dr. Ouhammou, Cadi Ayyad University Marrakesh, Morocco. Reference specimens of the *O. multicaulis* and *C. carvi* plants have been placed in the Faculty's herbarium, successively under the numbers MARK‐9834 and MARK‐9821.

### 4.2 Preparation of Extracts

Flowers of *O. multicaulis* and seeds of *C. carvi* were dried in laboratory in the shade and at room temperature. The dried materials were independently ground to powder. Subsequently, ethanol (600 mL) was added to 300 g of powder of each plant to obtain the extract using a Soxhlet apparatus at 75–79°C for 72 h. The ethanol was completely evaporated using a rotavapor, and the methanolic extracts were stored protected from light at 0–4°C. The concentrated extract weight of *C. carvi* was 40.2 g, representing a yield of about 13.4%, and 34.5 g of *O. multicaulis* extract, representing a yield of 18%.

### 4.3 Total Phenolic Compounds, Total Flavonoids, and Condensed Tannin Contents

The TPC, in the three methanolic extracts of *O. multicaulis*, *C. carvi*, and their mixture, was assessed using Folin–Ciocalteu method according to Singleton et al. [69] and Singleton and Lamuela‐Raventos [70] using GA as the standard. The extract of 100 µL was diluted with 3.7 mL of distilled water, and 200 µL of Folin–Ciocalteu reagent was added. After 3 min, 1 mL of a 20% sodium carbonate (Na_2_CO_3_) was added. The solution was then vortexed and incubated in dark for 45 min at 25°C. Then, absorbance was read at 765 nm against distilled water as a blank in a UV–Vis spectrophotometer (VR‐2000, Spain). The total phenolic concentration of methanolic extracts was calculated and expressed as mg equivalent GA equivalents per 100 g of the dry matter (mg of GAE/100 g DM) through the calibration curve of GA. Extractions were carried out in triplicate. The TFCs in the three methanolic extracts were estimated by aluminum trichloride method according to Zhishen et al. [71], using (+)‐catechin as the standard. Aliquots (200 µL) of diluted extract or (+)‐catechin standard solution were added to 60 µL of a 5% NaNO_2_ solution, followed by adding 40 µL of a 10% AlCl_3_, and incubated for 6 min before adding 400 µL of 1 M NaOH. Then, the mixture was brought to 1.2 mL by adding 500 µL of distilled water. The absorbance of the solution was measured immediately against the blank (the same mixture without the sample) at 510 nm in a UV–Vis spectrophotometer (VR‐2000, Spain). The TFCs in methanolic extracts were calculated and expressed as milligrams of (+)‐catechin equivalents per 100 g dry sample (mg of CAE/100 g DM), using the calibration curve of (+)‐catechin. The extraction was conducted in triplicate. TTC in the three methanolic extracts is evaluated following a slightly modified protocol of Xu et al. [72], using (+)‐catechin as the standard. Sample extract (300 µL) was added to a 3 mL of a 4% methanol vanillin solution and 1 mL of concentrated hydrochloric acid (HCl). After 15 min reaction, absorbance was measured at 500 nm against methanol as a blank in a UV–Vis spectrophotometer (VR‐2000, Spain). The amount of condensed tannin was calculated and expressed as mg catechin equivalents per 100 g dry matter (mg of CAE/100 g DM) using the calibration curve of (+)‐catechin. For each sample, triplicate extractions were performed.

### 4.4 HPLC Analysis

HPLC was used to identify and quantify phenolic compounds in the three extracts by using a Shimadzu HPLC System equipped with SCL‐10A pump, SIL‐10AD autoinjector, and an SPD‐10A UV/Vis detector (200–700 nm) (Shimadzu Japan), and Shimadzu data software was used. Reversed‐phase (RP‐18) columns (250 mm, 4.6 mm, 5.0 µm) (Agilent Technologies, Santa Clara, California, USA) were used for the separation, carried out at 25°C with an isocratic elution of acetonitrile (5%) and water (95%) in a constant flow rate of 0.1 mL min^−1^, injection volume of 10 µL, and phosphate buffer solution at pH 2.6. Analytical standards with typical retention times (caffeic, cinnamic, ferulic, gallic, *p*‐hydroxybenzoic, quinic, syringic, and vanillic acids; apigenin; catechin; luteolin; kaempferol; and quercetin) were used and compared to peaks of extracts of phenolic compounds [73].

### 4.5 Animals

Male Swiss mice (25–30 g) were provided by the Faculty of Science, Marrakesh animal house. They were housed in plastic boxes at room temperature (21°C ± 2°C) under a 12:12 h light/dark cycle with free access to food and water.

### 4.6 Drugs

All drugs were purchased from Sigma‐Aldrich and were prepared immediately before the experiments. The intraperitoneal (i.p.) injections were conducted with a volume of 10 mL kg^−1^ body wt. Each medication involved was solubilized in appropriate solvent as shown below: formalin (2%) and acetic acid (0.6%) in distilled water, herbal extracts, ASA, morphine sulfate (Actiskenan), and indomethacin were dissolved in saline solution (0.9% w/v).

### 4.7 Acute Toxicity Study

The acute toxicity test was performed on groups of six Swiss mice weighing between 17 and 25 g. The methanolic extracts of *C. carvi* and *O. multicaulis* were administered orally at the doses of 0.5, 1, 2, 3, 4, and 5 g kg^−1^ body wt. Control consisted of injections without extracts (0 g kg^−1^ body wt.). Mice were observed daily and for up to 14 days after administration to detect signs of toxicity and possible mortality [27, 28].

### 4.8 Selection of Doses

In order to determine effective and ineffective doses, different doses of *C. carvi* and *O. multicaulis* extracts, 100, 250, 500, 600, and 800 mg kg^−1^ body wt., were administered to laboratory animals. Minimum effective dose of each extract was selected for use as a primary treatment group, and non‐effective dose of each extract was chosen for combination into a mixture extract.

### 4.9 Antioxidant Assay In Vitro

#### 4.9.1 DPPH Activity

The free radical scavenging activity of the methanolic extracts was determined by the stable radical (DPPH), according to method described by Youssef et al. [74]. Briefly, 1.0–10 mg mL^−1^ of each extract was added to 2 mL of 60 µM methanol solution of DPPH and incubated at ambient temperature in obscurity. After 30 min, the absorbance was recorded against methanol as a blank at 517 nm. Quercetin and BHT were used as positive controls. The concentration of the extracts that neutralizes 50% of DPPH (IC_50_) was estimated using the following formula:

$$I\left(\%\right)=\left[\left(C\;-\;E\right)/C\right]\times 100$$

where *C* is the absorption of the control at 30 min. *E* is the absorption of the extract samples after 30 min.

#### 4.9.2 FRAP Test

The chelating capacity of extracts was determined by inhibition of the formation of the Fe (II)‐Ferrozine complex after incubation of the samples with the divalent iron according to the method described by Oyaizu et al. [75]. The method was based on the chemical conversion reaction of Fe^3+^ to Fe^2+^. Briefly, the sample and control substance were mixed with phosphate buffer (2.5 mL, 0.2 M, pH 6.6) and potassium ferricyanide (K_3_[Fe(CN)_6_]) (2.5 mL, 1%). A period of 30 min later, trichloroacetic acid was added (2.5 mL of 10% [w/v]). The mixture thus obtained was centrifuged 650 × *g* for 10 min. Finally, the upper layer was mixed with 2.5 mL distilled water and 0.5 mL of FeCl_3_ (ferric chloride 1%). The absorbance was measured at 700 nm after 15 min time of reaction. Quercetin and BHT were used as positive controls. Three replications were performed to calculate the mean value of the IC_50_.

### 4.10 Analgesic Tests (Pain Assessment)

To evaluate the possible analgesic effects of methanolic extracts, three animal experiments were performed: writhing, hot plate, and formalin tests. In this study, mice were divided into five groups of six each.

#### 4.10.1 Writhing Test

This test was selected as a model of acute peritoneal and visceral pain and conducted according to Deng et al. [76]. All animals received 0.1 mL of 0.6% acetic acid in saline, which was administered intraperitoneally. Treatments with distillated water (negative control), methanolic extracts of *C. carvi* (500 mg kg^−1^ body wt., p.o.), methanolic extracts *of O. multicaulis* (500 mg kg^−1^ body wt., p.o.), methanolic extract of the mixture (250 mg kg^−1^ body wt. of *O. multicaulis* and 250 mg kg^−1^ body wt. of *C. carvi* p.o.), and ASA (100 mg kg^−1^ body wt., i.p.) were administered 30 min before the injection of acetic acid to the groups of mice. The number of writhes in each group was counted during 30 min after the injection of acetic acid. The percentage inhibition of writhing reflex was calculated by the following formula:

%Inhibition=[(WRc−WRt)/WRc]×100

where WRc is the mean of writhe count in the negative control, and WRt is the mean of writhe count in methanolic extracts or ASA‐treated animal's groups.

#### 4.10.2 Hot Plate Test

In order to study the possible antalgic effect of methanolic extracts of *O. multicaulis*, *C. carvi*, and their mixture in animals submitted to a thermal stimulus, hot plate test was used a model of supraspinal analgesia according to Laughlin et al. [77]. The animals were treated with distillated water (control group), morphine (10 mg kg^−1^ body wt., i.p.), *O. multicaulis* extract (500 mg kg^−1^ body wt., p.o.), *C. carvi* extract (500 mg kg^−1^ body wt., p.o.), and the extract obtained from a mixture consisting of 250 mg kg^−1^ body wt. of *O. multicaulis* and 250 mg kg^−1^ body wt. of *C. carvi* p.o. All mice were then placed on a hot plate at 54°C ± 0.1°C. The latency time for the mouse on the hot plate without licking one of its paws or flicking its hind limb or jumping was considered the response time. A maximum time of 20 s was set up to prevent alteration of the mice's paws. The latency time in seconds for each treatment was compared with control.

#### 4.10.3 Formalin Test

Formalin test [78] was performed according to Hunskaar and Hole [30] and Wu et al. [79]. A volume of 20 µL of a 2% formalin solution in 0.9% NaCl was subcutaneously injected into the sub‐plantar surface of right paws in all mice using a microsyringe. The nociceptive response was recorded as the total amount of time spent licking the formalin‐injected paw. Two distinct periods of intensive licking activity were identified: The response was measured 0–5 min after the injection of the formalin solution (early phase or neurogenic phase) and 15–30 min after the injection (late phase or inflammatory phase). Thirty minutes prior to formalin injection, mice were divided into six groups (*n* = 6) and pretreated with vehicle (control group), morphine (10 mg kg^−1^ body wt., i.p.), *C. carvi* extract (500 mg kg^−1^ body wt., p.o.), *O. multicaulis extract* (500 mg kg^−1^ body wt., p.o.), and the extract obtained from a mixture consisting of 250 mg kg^−1^ body wt. of *O. multicaulis* and 250 mg kg^−1^ body wt. of *C. carvi* p.o.

#### 4.10.4 Tail Immersion Test

Tail immersion method [80] was also used to evaluate the central mechanism of analgesic activity produced by thermal stimulus, that is, by dipping the tip of the tail in hot water. Mice groups consisted of negative control: mice treated with distillated water, mice treated with methanolic extracts of *C. carvi* (500 mg kg^−1^ body wt., p.o.), mice treated with methanolic extracts of *O. multicaulis* (500 mg kg^−1^ body wt., p.o.), mice treated with methanolic extract of the mixture (250 mg kg^−1^ body wt. of *O. multicaulis* and 250 mg kg^−1^ body wt. of *C. carvi* p.o.), and mice treated with SA (100 mg kg^−1^ body wt, i.p.). Thirty minutes after the extracts and SA administration, the rats were placed in contortion cylinders with the animal's tail immersed up to 5 cm in hot water at a temperature of 55°C. A maximum latency period of 20 s was considered to avoid tail tissue damage in mice. The flick response was evaluated by measuring the latency time it takes the animal to withdraw its tail using a stopwatch (in seconds).

### 4.11 Anti‐Inflammatory Study by Xylene‐Induced Ear Edema Test

To evaluate in vivo anti‐inflammatory activity, extracts were evaluated in xylene‐induced mouse ear edema. The study was conducted by xylene‐induced ear edema test according to the method of Xu et al. [81]. Five groups (*n* = 6) were used. Treated mice group received methanolic extracts at 20 mg kg^−1^ body wt. p.o. through oral administration. Reference group received indomethacin (20 mg/kg body wt.). Thirty minutes after treatments, edema was induced by applying 20 µL xylene into the anterior surface of the right ear while the left ear was considered control. After 15 min of xylene application, all animals were anesthetized with chloral hydrate (6% i.p.) and the two ears were clipped, dimensioned, and weighed. Ear edema was calculated by subtracting the weight of the untreated left ear section from that of the treated right ear section (average weight difference between the right and left ears for each group), and the percentage of edema inhibition was determined following the equation:

$$\mathrm{Inhibition}\;\mathrm{of}\;\mathrm{edema}\left(\%\right)=[(\mathrm{Wc}\;-\;\mathrm{Wt})/\mathrm{Wc}]\times 100$$

where, Wc is the difference of ear weight in the control group, and Wt is the difference of ear weight in the test group.

### 4.12 Statistical Analysis

Statistical analyses were performed using Sigma‐Plot software (version 13). All results were reported as mean ± SEM. The comparison between different groups was performed by use of one‐way ANOVA analysis that was followed by Tukey's test. A value of *p* < 0.05 was regarded as statistically significant.

## Author Contributions

**El Mahdi Wakrim**: conceptualization, methodology, investigation, writing – original draft preparation. **Brahim Bouizgarne**: conceptualization, software, validation, formal analysis, data curation, writing – original draft preparation, writing – review and editing, visualization, supervision. **Hamid Kabdy**: conceptualization. **Abderrahman Chait**: conceptualization, validation, resources, writing – original draft preparation, writing – review and editing, supervision, project administration, funding acquisition. **Sami Mnif**: methodology, writing – review and editing. **Mehdi Ait Laaradia**: methodology, software, writing – original draft preparation. **Jawad Laadraoui**: software, writing – original draft preparation. **Karima Raoui**: software. **Chafik Terrafe**: software. Stefania Garzoli, writing – review and editing, supervision. All authors have read and agreed to the published version of the manuscript.

## Ethics Statement

All experiments were performed in compliance with the guidelines of the European Community (Directive 86/609/EEC, 24.11.1986). Every effort was made to minimize the number of animals used in all experiments. Acute toxicity assessment followed standard procedures described in OECD Guideline 425 issued in 2008. Approval number was not required or assigned.

## Conflicts of Interest

The authors declare no conflicts of interest.

## Data Availability

All the data in the article are available from the corresponding author upon reasonable request.
